# A quantitative test of the face validity of behavior-change messages based on the Brazilian Dietary Guidelines

**DOI:** 10.1186/s12937-021-00668-7

**Published:** 2021-01-26

**Authors:** Neha Khandpur, Fernanda Paranhos Quinta, Patricia Constante Jaime

**Affiliations:** 1 Department of Nutrition, School of Public Health, University of São Paulo, Av. Dr. Arnaldo, 715-Cerqueira César, São Paulo, 01246-904 Brazil; 2 Center for Epidemiological Studies in Health and Nutrition (NUPENS), Faculty of Public Health, University of São Paulo, Av. Dr. Arnaldo, 715-Cerqueira César, São Paulo, 01246-904 Brazil; 3 Department of Nutrition, Harvard T.H. Chan School of Public Health, 677 Huntington Avenue, Boston, MA 02115 USA

**Keywords:** Behavior-change messages, Dietary guidelines, Face validity, Motivating potential, Participant receptivity, Brazil

## Abstract

**Background:**

Implementation science has scant evidence of how dietary guidelines can be developed into actionable behavior-change messages and even less evidence on their motivating potential and perceived effect on behavior. This may explain the widening gap between nutrition science and individual behavior and the low uptake of dietary recommendations by the population for which they are intended. This study aimed to: (i) assess participant receptivity and acceptance of behavior-change messages; (ii) determine if the behavior-change strategies used in the messages and the main theme they relayed influenced participant evaluation of the messages; (iii) explore if evaluations varied by participants’ stage of behavior-change; and (iv) elucidate reasons for non-compliance with the messages.

**Methods:**

An online survey was used to test the face validity and participant receptivity of 28 behavior-change messages, among a diverse sample of 2400 adult Brazilians. Participants’ understanding of the messages, message likeability and convincingness, and the probability that participants would change behavior in accordance with the message were measured, along with reasons for non-compliance.

**Results:**

The mean overall scores suggested that participants liked the messages, understood them, and found them convincing. As expected, the probability of complying with the messages scored lower compared to other study outcomes. Messages about shopping practices, cooking practices, and planning and organization performed better than those on other themes. Participants were more receptive to messages that included behavior-change strategies like goals, social identity, and pleasure, however, the probability of compliance was higher for messages with constructs that emphasized health and cost consequences. Participants trying to change their diet or seeking resources to support healthier dietary choices had greater engagement with and receptivity to the messages. Time and effort, and high costs associated with making healthy changes, were barriers to compliance.

**Conclusions:**

Messages may help improve individual understanding, stimulate interest in a topic and get participants engaged, particularly if messages are goal-oriented and highlight the pleasure and collective identity of these food-related behaviors. However, messages stop short of addressing the structural, social, and economic barriers to healthy diets. These aspects will need to be targeted through legislative action for sustainable behavior change.

**Supplementary Information:**

The online version contains supplementary material available at 10.1186/s12937-021-00668-7.

## Contributions to the literature

This study:

1. Quantitatively tests participant receptivity and acceptance of behavior-change messages, adding to the scant literature on the role of messaging in supporting behavior change.
2. Details the methodology of the development of the data collection tool, and the indicators used to assess participant evaluation of messages – participant understanding, message likeability, and participant probability of changing behavior.
3. Presents analysis by the different themes of the messages as identified in the 2014 Brazilian Dietary Guidelines and also by the varying effectiveness of the theory-based, behavior-change strategies that were incorporated into the messages.
4. Captures the reasons for participant non-compliance with the messages and reflects on the limits of messaging as a driver of behavior-change.

## Introduction

Effective, actionable messages are central to public health campaigns and health promotion initiatives. Several health promotion efforts that focus on some form of behavior-change, like smoking cessation [1], are frequently coupled with a messaging component that is delivered in printed form like brochures or, increasingly, through electronic means like short message service (SMS), to support and reinforce healthy behaviors [2, 3]. Dietary guidelines, a public health tool based on nutrition science that provides advice on healthy food choice, is an example of an initiative in need of behavior-change messaging for effective dissemination and uptake. While the dietary recommendations have become increasingly evidence-based, their limited uptake by the population for which they are intended [4], points to a widening gap between nutrition science and population behavior. Behavior-change health messages could help close this knowledge-behavior gap by translating the scientific and technical content of the dietary guidelines into messages about specific practices that the general public can better understand and implement.

Beyond relaying information, messages need to engage, convince, and motivate their audience to modify their dietary behavior. To improve their likelihood of doing so, it is recommended that messages incorporate key elements of health communication, including both theory-based and audience-centric strategies. Messages could therefore be constructed to target individual factors like self-efficacy [5], or be framed to highlight the benefits of modifying behavior (gain-framed) [6], or present strategies to overcome barriers to adopting healthy behavior [7]. Identifying the stage of behavior change of the intended audience as described by the Transtheoretical Model [8], and tailoring the messages to address the stage-based needs has also been recommended [9]. Indeed, existing evidence supports the use of tailored, gain-framed, and self-efficacy-based messaging for preventive behaviors [10–12], and suggests that a combination of these approaches and techniques may be most effective in supporting dietary behavior-change.

Once messages have been constructed, pretesting messages to assess how well they are understood by their target population is the crucial next step in message development. Few studies have documented the process of message development based on dietary guidelines [13, 14] and fewer still have quantitatively tested the motivating potential and perceived effect of these messages on behavior [15, 16]. There is also a clear need for more evidence on what theoretical constructs incorporated in the behavior-change messages are effective at encouraging healthy dietary behavior. This study proposes to gauge participant receptivity and acceptance of behavior-change messages among a sample of the adult population for whom these messages were designed. The messages were based on information from the 2014 Dietary Guidelines for Brazilians and incorporated key concepts from health communication and health promotion.

The study objectives are to:

1. Assess participant receptivity and acceptance of behavior-change messages, as measured by participant evaluations of message understanding, likeability, potential to convince and the probability of complying with the messages;
2. Determine if the main theme relayed by the message and the behavior-change strategies used in the development of the messages influenced participant evaluations;
3. Explore if participant assessment of the messages varied by their stage of behavior-change; and
4. Better understand the barriers to changing behavior faced by participants and their reasons for non-compliance with the messages.

## Methods

### Study design, study sample and recruitment

The face validity of 28 messages, gauged by participant receptivity and acceptance, was tested in May – June of 2018 (Supplementary material). A Brazil-based survey firm, MindMiners, was contracted to help recruit adult participants for the study. The MindMiners platform was used to develop the survey instrument which was then distributed to 2400 adult Brazilians who were part of the online panel of participants and had consented to being involved in the study. The participants in the study sample were 18 years or older, with varying educational attainments, SES levels, and employment status and were recruited to represent different regions of Brazil (see Table 1). They were invited to give their feedback to a series of messages. They did not receive any reimbursement from the study team for participating in the study. All study procedures were approved by the Ethics Committee of the University of São Paulo (69039117.0.0000.5421) and were conducted in Portuguese.

**Table 1:** Study demographics

Indicators	Total sample, ***N*** = 2400
Age, mean yrs. (SD)	28.79 (8.48)
Sex, %	
Female	55.33
Male	44.67
Education, %	
Primary or less	8.71
Secondary	50.29
Tertiary	41.00
SES, %	
Low	11.00
Medium	73.46
High	15.56
Employment status, %	
Employed	52.17
Student	18.25
Unemployed	26.71
Retired	2.88
Region, %	
South east	50.83
North east	23.04
South	15.21
Central western	6.79
North	4.13

### Development of the behavior-change messages

The details of message development and refinement have been presented in a separate manuscript [17]. Briefly, a sequential, five-step, mixed-methods approach was undertaken prior to this study that included: (i) content extraction of 63 excerpts from the 2014 Brazilian Dietary Guidelines; (ii) a secondary analysis of the literature to highlight barriers to healthy eating among adult, urban-dwelling Brazilians who were the intended audience of these messages (audience analysis); (iii) input from a 4-member expert review panel to help prioritize the excerpts for message development; (iv) the development of 111 messages by the research team, guided by the use of the Theoretical Domains Framework [18], and; (v) a test of the content validity of the messages conducted among 36 researchers across Brazil who had contributed to the development of the Dietary Guidelines or used it in their current research, on the clarity and importance of the messages. At the end of this phase 40 messages were shortlisted. For the purposes of this study it was important that respondents were presented with messages of a consistent theme (see below), and length (where possible) to be suitable for dissemination via an electronic platform. Accordingly, within themes a few related messages were combined and some messages with very close substitutes discarded. This resulted in a final sample of 28 messages (Supplementary material).

### Message content and attributes

The messages for this study were developed to inform the adult Brazilian population of specific activities and choices related to food that would help them more closely align their everyday behaviors with the recommendations from all five chapters of the 2014 Brazilian Dietary Guidelines. This required multiple messages to be crafted, with the objective of being disseminated collectively, in a staggered manner or simultaneously, in combination with future behavior-change interventions. Messages were designed to be delivered via text message or social media campaigns, with studies suggesting some benefit of these dissemination platforms for behavior change [19].

#### Message themes

Message content focused on one of five themes: (i) the categorization of food based on the NOVA (not an acronym) classification, (ii) choices and best practices related to planning and organizing meals, (iii) shopping for food, (iv) cooking food, or (v) eating food. The NOVA classification categorizes food items into mutually exclusive categories differentiated by the level and extent of industrialized processing that they undergo [20]. It considers the physical, biological, and chemical methods used during the manufacturing process.

Messages about the NOVA classification (*n* = 8) introduced the categories of *unprocessed or minimally processed food*, *processed food,* and *ultra-processed food* [20]. Messages on planning and organization focused on meal planning and organizing the pantry (*n* = 2), and those on shopping highlighted the best places to shop at and the kinds of foods to purchase (*n* = 2). Cooking with the family, division of labor, and advice on condiments and forms of cooking were relayed by messages on the theme of cooking (*n* = 7). Finally, messages on the theme of eating focused on choices of snacks and meals eaten at home or in restaurants, and on the eating ambience (*n* = 9).

#### Message constructs

Theory-informed strategies or constructs were incorporated into the messages [17]. These included providing substitutions for, or solutions to, the barriers associated with planning and organizing healthy meals, or shopping or cooking; they highlighted the health benefits, cost gains, gender equity or environmental benefits of the behaviors they were promoting. Skills, goals, social identity, and eating as a source of positive emotion (pleasure) were the constructs that were informed by the Theoretical Domains Framework [18]. Messages were short, simple, direct, and positively framed (gain-framed) [21, 22].

### Study procedures and the study instrument

To allow for detailed participant feedback on a subset of messages and to account for logistical limitations of the survey platform, it was not possible to show all participants all messages. Instead, messages  were divided into 3 groups, with messages of the same theme presented together. Participants were randomly assigned to a group in a 1:1:1 ratio. This ensured that the three separate groups of participants would, on average, have comparable characteristics and comparable feedback on the messages, and be representative of the original sample of 2400 participants from which they were drawn.

Participants in group 1 saw 10 messages (8 focused on the NOVA classification, 2 on planning). Nine messages were evaluated by group 2 (2 on grocery shopping, 7 on cooking) and 9 messages were assessed by group 3 (all on the theme of eating). Across all 28 messages, the construct of having a goal was represented in 15 messages, developing skills was the focus of 11 messages, solutions for barriers to healthy eating were provided by 12 messages, while substitution towards healthier practices (*n* = 9), health benefits (*n* = 6), cost benefits (*n* = 4), environmental benefits (*n* = 3), pleasure (*n* = 4) and social identity (*n* = 2) were also incorporated into these messages.

Once participants were randomly assigned to a group, they responded to a 3-part survey. The first part asked questions about participant food-related behaviors and perceptions – their current diet, the perceived healthfulness of their current food practices, factors that most influence their food purchase and consumption choices, and their openness to making healthier food choices (see Table 2). The second part of the survey focused on obtaining feedback on the messages. Messages were presented one at a time and, for each message, participants had to rate, on a scale of 1–7, statements that captured the ease of understanding of the message [*This message is easy to understand*], their likeability of the message [*I like this message*], how convincing they thought the message was [*This message is convincing*] and how likely they were to comply with what the message was relaying [*How likely are you to change your current behavior to follow what the message recommends?*]. Participants were also asked to indicate their reasons for not wanting to comply with the message [*What are some reasons why you would not follow the message? You can choose more than one option*. Response options: *No interest in topic; Not responsible for activity at home; Don’t really understand message; Appears expensive to follow; Appears to require a lot of time and effort; Benefits seem exaggerated*]. The third and final part of the survey collected participant demographic information.

**Table 2:** Food-related behaviors and perceptions of the study sample

Current dietary behaviors and perceptions	Total sample, ***N*** = 2400
**Current diet,** %	
Omnivore	84.04
Pescatarian	4.58
Ovo-lacto vegetarian	8.58
Vegan	2.79
**Current food practices,** %	
I do not usually think much about my food choices	18.33
I sometimes think about how to improve my food choices	43.96
I’m currently making changes towards healthier food choices and I am well informed about nutrition guidelines	23.50
I actively make decisions to improve my food choices and I am always looking for more guidance	14.21
**How healthy do you consider your current eating habits?** (1 very unhealthy – 7 very healthy), Mean (SD)	4.15 (1.39)
**Factors that most influence food purchases or consumption,** %	
Cost	71.67
Quality – freshness, safety	66.71
Time spent in food preparation	28.13
Effect on health	44.17
Taste	58.33
Environmental impact	11.17
**Opinions about making healthier food choices,** %	
Frankly, have a healthier diet doesn’t matter to me	9.63
I am open to achieving this goal, as long as it is practical and easy to follow	31.25
I am open to achieving this goal, as long as it does not require much time or money	36.75
I would definitely like to improve my food choices by all means necessary	22.38

### Data analysis

The main study outcomes were participants’ understanding of the messages, likeability and message convincingness, and the probability that participants would change behavior in accordance with the message. The data from 2400 participants were downloaded as an Excel file and imported and analyzed in long form to account for co-variation in participant responses across all the messages from the same group that they provided feedback for, in STATA v.14. Since only completed responses were shared by the contracted survey firm, no further data cleaning was necessary.

Descriptive analyses were used to capture mean participant feedback. Linear regression was used to capture differences in main study outcomes by the theme of the message and by message constructs. Multivariate analyses were used to assess the independent effects of themes and constructs on study outcomes. Exploratory univariate linear regression models were used to examine if responses to study outcomes varied by food-related behaviors and perceptions of the participant as captured in the first part of the survey.

## Results

The mean age of the sample was 28.79 years (± 8.48), a majority of the sample had completed secondary or tertiary education, had medium or higher SES levels and was employed (see Table 1). Balance tests indicated no significant differences between groups for all indicators except educational attainment and geographic representation, where very small substantive differences were seen. Controlling for these variables in later analysis did not change the study results.

A majority of this study sample self-reported as being omnivore (84%). Participants considered their current eating habits to be relatively healthy with a mean score of 4.15 out of 7 (7 being very healthy). While over 40% sometimes thought about how to improve the healthfulness of their diets, only about 23% were actively making changes and felt well informed about the dietary recommendations available. Less than 15% were seeking more guidance in this regard (see Table 2). Cost was the number one factor determining food purchase and consumption decisions in this sample, followed by food quality and taste. Environmental concerns factored into food choices for one in nine participants. In general, the sample reported being open to making healthier choices if these choices did not require too much time, were not expensive or if they included advice that was easy to follow. Only about one in five participants was open to making changes to their food choices by all means necessary.

Overall, participants scored the messages highly on all indicators. The mean score for message likeability was 5.47 on a scale of 1–7, message ease of understanding was 5.67, and the message’s potential to convince was scored at 5.34. The likelihood of complying with the message was scored relatively lower at 4.70.

When examining scores across message themes in the univariate analyses (Table 3), messages about grocery shopping practices scored significantly higher on likeability compared to the messages about the NOVA classification. Messages about cooking practices were easier to understand and those on planning were more convincing and more likely to be followed. Messages that were goal-based or included the construct of solution or substitution scored lower than those without this construct for likeability, convincingness, and probability of complying with the message, but higher on ease of understanding. Including constructs of skill, pleasure and health consequences in the messages increased scores on all outcomes, although there was no difference in ease of understanding for health consequences. Including cost consequences or environmental consequences helped improve scores on likeability and message convincingness, but scores on understanding and likelihood of complying with the message recommendations were lower for the construct of environmental consequences (Table 3).

**Table 3:** Participant feedback on the messages, univariate analysis

Message themes and strategies	Likeability		Ease of understanding		Convincingness		Likelihood of complying	
	(1 totally disagree/very improbable – 7 totally agree/ very probable), Mean (SE)							
Overall	5.47 (0.01)		5.67 (0.01)		5.34 (0.01)		4.70 (0.01)	
	Beta	Score	Beta	Score	Beta	Score	Beta	Score
NOVA classification	Ref	5.43 (0.01)	Ref	5.42 (0.02)	Ref	5.38 (0.01)	Ref	4.54 (0.03)
Planning	0.18*	5.61 (0.03)	0.29*	5.71 (0.04)	0.16*	5.55 (0.03)	0.37*	4.91 (0.04)
Shopping	0.28*	5.71 (0.03)	0.21*	5.63 (0.04)	0.04	5.42 (0.04)	−0.03	4.51 (0.04)
Cooking	0.14*	5.57 (0.01)	0.42*	5.84 (0.02)	0.09*	5.48 (0.02)	0.23*	4.78 (0.02)
Eating	−0.08*	5.34 (0.01)	0.34*	5.76 (0.02)	−0.22*	5.16 (0.01)	0.16*	4.71 (0.02)
Goals not used	Ref	5.52 (0.01)	Ref	5.64 (0.01)	Ref	5.41 (0.02)	Ref	4.77 (0.02)
**Goals used**	−0.10*	5.42 (0.01)	0.06*	5.70 (0.01)	−0.12*	5.29 (0.01)	−0.13*	4.64 (0.01)
Skills not used	Ref	5.41 (0.01)	Ref	5.62 (0.02)	Ref	5.29 (0.01)	Ref	4.67 (0.02)
**Skills used**	0.15*	5.57 (0.02)	0.12*	5.75 (0.02)	0.15*	5.44 (0.02)	0.07*	4.74 (0.02)
Solution not used	Ref	5.47 (0.01)	Ref	5.60 (0.01)	Ref	5.36 (0.01)	Ref	4.70 (0.01)
**Solution used**	−0.00	5.46 (0.01)	0.15*	5.76 (0.02)	−0.03	5.32 (0.02)	−0.00	4.70 (0.02)
Substitution not used	Ref	5.51 (0.01)	Ref	5.61 (0.15)	Ref	5.41 (0.01)	Ref	4.71 (0.02)
**Substitution used**	−0.12*	5.39 (0.01)	0.14*	5.76 (0.02)	−0.17*	5.24 (0.02)	−0.02	4.69 (0.02)
Health consequences not used	Ref	5.45 (0.01)	Ref	5.66 (0.01)	Ref	5.32 (0.01)	Ref	4.68 (0.01)
**Health consequences used**	0.07*	5.53 (0.2)	0.03	5.70 (0.03)	0.12*	5.45 (0.03)	0.11*	4.79 (0.03)
Cost consequences not used	Ref	5.44 (0.01)	Ref	5.66 (0.01)	Ref	5.31 (0.01)	Ref	4.70 (0.01)
**Cost consequences used**	0.18*	5.62 (0.02)	0.03	5.69 (0.03)	0.21*	5.51 (0.02)	−0.01	4.69 (0.03)
Environmental consequences not used	Ref	5.45 (0.01)	Ref	5.67 (0.01)	Ref	5.33 (0.01)	Ref	4.72 (0.01)
**Environmental consequences used**	0.21*	5.66 (0.03)	− 0.04	5.62 (0.04)	0.11*	5.44 (0.03)	−0.18*	4.54 (0.04)
Social identity not used	Ref	5.37 (0.00)	Ref	5.67 (0.01)	Ref	5.34 (0.01)	Ref	4.71 (0.01)
**Social identity used**	0.05	5.52 (0.05)	−0.02	5.65 (0.07)	0.11	5.46 (0.06)	−0.12	4.59 (0.07)
Pleasure not used	Ref	5.46 (0.01)	Ref	5.66 (0.01)	Ref	5.33 (0.01)	Ref	4.67 (0.01)
**Pleasure used**	0.09*	5.56 (0.03)	0.18*	5.84 (0.04)	0.09*	5.43 (0.04)	0.36*	5.04 (0.05)

*Significantly different, *p*-value < 0.05

Multivariate analysis that controlled for other message constructs and themes confirmed some of the findings from the univariate analysis. Compared to the NOVA-themed messages, those on shopping practices scored higher on message likeability, holding all other message attributes constant. Messages on cooking practices scored higher on ease of understanding and on message convincingness and messages on planning received higher scores on the likelihood of complying (Table 4), messages that were goal-based had higher scores across all outcomes except likelihood of complying with the message. Likeability scores increased for the constructs of skill and social identity, but likeability scores were lower for the constructs of solution, substitution, cost, and environmental consequences. Scores on ease of understanding of the messages only improved for the construct of social identity. Message convincingness increased for the construct of health consequences and decreased for substitution. Scores for the likelihood of complying with the message increased with the constructs of pleasure, substitution, and solution, cost and health consequences but decreased with skills and goals.

**Table 4:** Participant feedback on the messages, multivariate analysis

Message themes and strategies	Likeability		Ease of understanding		Convincingness		Likelihood of complying	
	(1 totally disagree/very improbable – 7 totally agree/ very probable), Mean (SE)							
	Beta	Score	Beta	Score	Beta	Score	Beta	Score
NOVA classification	Ref	5.28 (0.03)	Ref	5.30 (0.04)	Ref	5.32 (0.03)	Ref	4.58 (0.05)
Planning	0.22*	5.51 (0.06)	0.59*	5.89 (0.07)	0.26*	5.58 (0.07)	0.60*	5.18 (0.08)
Shopping	0.84*	6.12 (0.12)	0.64*	5.94 (0.14)	−0.03	5.29 (0.13)	−0.27	4.31 (0.15)
Cooking	0.39*	5.68 (0.03)	0.74*	6.04 (0.04)	0.29*	5.62 (0.04)	0.13	4.72 (0.05)
Eating	0.03	5.32 (0.02)	0.30*	5.61 (0.03)	−0.17*	5.15 (0.03)	0.14*	4.73 (0.04)
Goals not used	Ref	5.53 (0.02)	Ref	5.49 (0.02)	Ref	5.26 (0.02)	Ref	4.85 (0.07)
**Goals used**	0.18*	5.56 (0.02)	0.34*	5.84 (0.02)	0.14*	5.41 (0.02)	−0.27*	4.58 (0.06)
Skills not used	Ref	5.42 (0.02)	Ref	5.67 (0.03)	Ref	5.36 (0.02)	Ref	4.84 (0.05)
**Skills used**	0.13*	5.55 (0.03)	−0.01	5.66 (0.04)	−0.04	5.31 (0.04)	−0.33*	4.52 (0.06)
Solution not used	Ref	5.56 (0.02)	Ref	5.68 (0.02)	Ref	5.36 (0.02)	Ref	4.64 (0.03)
**Solution used**	−0.21*	5.35 (0.02)	−0.04	5.65 (0.03)	−0.04	5.32 (0.03)	0.12*	4.76 (0.03)
Substitution not used	Ref	5.53 (0.01)	Ref	5.69 (0.02)	Ref	5.38 (0.02)	Ref	4.57 (0.03)
**Substitution used**	−0.15*	5.37 (0.02)	−0.05	5.64 (0.03)	−0.11*	5.28 (0.03)	0.28*	4.86 (0.03)
Health consequences not used	Ref	5.46 (0.01)	Ref	5.67 (0.01)	Ref	5.32 (0.01)	Ref	4.62 (0.02)
**Health consequences used**	0.02	5.48 (0.03)	0.01	5.68 (0.03)	0.16*	5.48 (0.03)	0.38*	5.01 (0.05)
Cost consequences not used	Ref	5.48 (0.01)	Ref	5.68 (0.02)	Ref	5.32 (0.01)	Ref	4.65 (0.02)
**Cost consequences used**	−0.09*	5.38 (0.03)	−0.08	5.60 (0.05)	0.10	5.42 (0.04)	0.26*	4.91 (0.05)
Environmental consequences not used	Ref	5.50 (0.01)	Ref	5.68 (0.02)	Ref	5.33 (0.02)	Ref	4.67 (0.01)
**Environmental consequences used**	−0.35*	5.14 (0.12)	−0.17	5.50 (0.13)	0.10	5.44 (0.11)	0.28	4.96 (0.13)
Social identity not used	Ref	5.46 (0.01)	Ref	5.66 (0.01)	Ref	5.34 (0.01)	Ref	4.69 (0.01)
**Social identity used**	0.30*	5.76 (0.08)	0.23*	5.89 (0.11)	0.01	5.36 (0.09)	0.19	4.89 (0.11)
Pleasure not used	Ref	5.46 (0.01)	Ref	5.67 (0.01)	Ref	5.33 (0.01)	Ref	4.65 (0.01)
**Pleasure used**	0.03	5.50 (0.05)	0.00	5.67 (0.06)	0.11	5.44 (0.05)	0.63*	5.29 (0.06)

*Significantly different, *p*-value < 0.05

In exploratory analysis of differences in participant responses across different food-related practices, scores on all study outcomes of likeability, understanding and convincingness increased when participants reported actively making healthy food choices compared to being in a pre-contemplation or a contemplation stage where they were not thinking of, or only sometimes thinking about, their food choices. Similarly, participants who were willing to change current behaviors using all means possible had higher scores across all outcomes compared to participants for whom consuming a healthy diet was not a priority (Table 5).

**Table 5:** Variation in participant feedback across food-related practices and perceptions, univariate analysis

Messages	Likeability	Ease of understanding	Convincingness	Likelihood of complying
	(1 totally disagree/very improbable – 7 totally agree/ very probable), Mean (SE)			
**Current food practices**				
I do not usually think much about my food choices	4.97 (0.02) Ref	4.90 (0.03) Ref	4.95 (0.02) Ref	3.89 (0.03) Ref
I sometimes think about how to improve my food choices	5.41 (0.01) *	5.69 (0.02) *	5.25 (0.01) *	4.62 (0.02) *
I’m currently making changes towards healthier food choices and I am well informed about nutrition guidelines	5.69 (0.02) *	6.00 (0.02) *	5.57 90.02) *	5.09 (0.02) *
I actively make decisions to improve my food choices and I am always looking for more guidance	5.88 (0.02) *	5.97 (0.03) *	5.70 (0.03) *	5.31 (0.03) *
**Opinions about making healthier food choices**				
Frankly, have a healthier diet doesn’t matter to me	4.94 (0.03) Ref	4.80 (0.04) Ref	4.91 (0.04) Ref	3.92 (0.04) Ref
I am open to achieving this goal, as long as it is practical and easy to follow	5.03 (0.01) *	5.51 (0.02) *	5.22 (0.02) *	4.51 (0.02) *
I am open to achieving this goal, as long as it does not require much time or money	5.52 (0.01) *	5.87 (0.02) *	5.37 (0.02) *	4.79 (0.02) *
I would definitely like to improve my food choices by all means necessary	5.82 (0.02) *	5.91 (0.02) *	5.64 (0.02) *	5.15 (0.03) *

*Significantly different, *p*-value < 0.05

Among the reasons why participants reported not being likely to comply with message recommendations, the perceptions that the message would require a lot of time and effort, or financial resources, were most often selected. Not being responsible for the food related decisions or practices at home and disinterest in the topic were some other reasons reported (Table 6).

**Table 6:** Reasons why participants would not comply with the information contained in the messages

Reasons	% that chose this option
No interest in topic	4.78
Not responsible for activity at home	5.61
Don’t really understand message	1.69
Appears expensive to follow	7.06
Appears to require a lot of time and effort	7.97
Benefits seem exaggerated	2.75

## Discussion

This study assessed the face validity of messages based on the Brazilian Dietary Guidelines by assessing participant receptivity to behavior-change messages across dimensions of message likeability, understanding, potential to convince and the likelihood of complying with the message. It also sought to determine if message themes or behavior-change strategies used in message development positively influenced participant assessment. Overall, the messages were well received by the study participants. Messages which highlighted behavioral goals, social identity and health consequences were more favorably assessed.

The mean overall scores suggested that participants liked the messages, understood them, and found them convincing. As expected, the probability of complying with the messages scored lower compared to other study outcomes. The potential of dietary messages to convince and motivate has been previously tested with behavior-change messages based on the 2010 Dietary Guidelines for Americans [23]. While the messages in that study had a different thematic focus from the messages developed in the present study (their content was directed towards calories, physical activity, energy balance, portion sizes etc.), the general approach to message development was similar. Much like the current study, the messages scored relatively higher on their motivating potential than on their likelihood to change behavior [23]. These results suggest that messages based on dietary guidelines have a role to play in increasing general awareness and motivation, and in presenting best practices for adopting a healthier diet. However, additional interventions will be required for any long-term, sustainable behavior change.

Messages communicated content across different food-related activities, from introducing the NOVA classification, to best practices and healthy strategies related to planning, shopping, cooking, and eating. Differences revealed by the multivariate analysis demonstrated that participants liked messages on shopping practices, best understood and were most convinced by messages on cooking practices and were most likely to follow the messages on planning. In terms of ease of modifying current behavior, it seems plausible that participants would choose to start with the basics of planning and organization – these strategies may be perceived to be under the participant’s control, possibly requiring less systemic change within and outside the household or involving input or support from fewer family members to implement. This also seems like a positive first step for changing other behaviors as well, since planning is fundamental to shopping, cooking, and eating.

Messages evaluated in this study also incorporated constructs of skills, goals, social identity, and eating as a source of pleasure; they emphasized solutions to common barriers or substitution with a healthier behavior, and stressed the health, cost or environmental consequences. Among these constructs, likeability of messages was significantly improved when the construct of social identity was used in the message, followed by messages that articulated a goal or focused on developing a skill. Messages with a social identity component were also well understood, followed by goal-focused messages. Highlighting the health benefits and a goal-focus seemed to better convince participants, who scored them higher than other message constructs. In terms of likelihood of complying with the message, highlighting the pleasure participants could derive from following the message seemed to work best for this sample, followed by health and cost consequences. These results suggest that communicating certain constructs within behavior-change messages, like having a set of qualities associated with a collective identity or having clear objectives to achieve, makes participants more receptive to them. In terms of probability of compliance with a message though, more practical and utilitarian aspects, like impact on health and costs, sway participants. Pleasure was also a key motivator for intended action. These findings find support in published literature. In an online study by Vaillancourt et al., messages using pleasure-based and health-based approaches appeared to be equally persuasive and believable in their sample of Canadian adults [24]. Results from their study suggest a clear role that communication strategies emphasizing a pleasure-oriented approach can play, beyond the health-oriented approach, to foster healthy eating habits. Besides positive message appraisals, both these messaging approaches have also been shown to lead to healthier meal choices among participants with lower diet quality scores [25].

If the participant was making an effort to change their diet at the time of the study, it resulted in greater engagement with and receptivity to the messages. Participants seeking resources to support healthier dietary choices also evaluated messages more favorably. Both current food practices and openness to making healthier food choices reflected participants’ stage of change as described by the Transtheoretical Model of health behavior change [8] – the closer they were to the action stage (making overt changes), the more convinced they were by the messages and the more likely they were to comply. For participants in the action and maintenance stages, focusing on the advantages in dietary behavior change has been shown to be advantageous by a meta-analytic review [26]. The study sample had already identified as being cost-sensitive and so it was not surprising to find time and effort, or cost associated with making healthy changes, held participants back from complying with the messages. Time costs, convenience (or the lack thereof) and financial costs have been well defined barriers to healthy eating in the literature [27, 28]. Defined roles and responsibilities around food choices and eating were other barriers to compliance that were highlighted by this study.

Certain limitations of the study are worth highlighting. Only perceptions and opinions of participants were captured in this study to gauge general receptivity to the messages. As next steps, analysis of the actual behavior-change potential of these messages is warranted. Messages were evaluated online, not in the presence of a researcher, so while social desirability in the responses may be less of a concern, it may be difficult to generalize these results to other channels of communication that could be used to deliver these messages. Other strategies in message development like message tailoring based on users’ physiological or psychological states has found some support in health communication and promotion fields [29]. While messages in this study accounted for general psychological barriers among Brazilians, messages were not tailored to specific participant needs. Strengths include the regional and socio-economic diversity of the sample, the multiple relevant study outcomes for which information was collected, and the messages themselves – message development kept the target audience in mind and underwent a rigorous and transparent review and revision that included a panel of experts, which resulted in their positive reception.

## Conclusion

This study found high acceptance of the messages within this diverse sample of adult Brazilians. However, participant responses also highlighted that messages can only go so far in stimulating behavior-change towards healthier diets, especially among those not contemplating changing their food choices. Messages may help address some of the determinants of food choice operating at the level of the individual. They could help improve knowledge and awareness, stimulate interest in a topic and get participants engaged. However, behavior is a product of individual and collective action, and is influenced by the structural, social, and economic context [30, 31]. Therefore, in combination with awareness-raising and motivating messages, changing human behavior must address the powerful social, political, and economic barriers to healthy diets through legislative action [32]. Despite their appeal and ability to inform and increase awareness, these messages are the very first step to behavior change and are intended to be used in conjunction with other initiatives. The individual effort required to circumvent affordability, access, availability, and marketing of unhealthy products is overwhelming and, unless regulated, these environmental determinants will continue to be a deterrent to healthy choices.

## Supplementary Information

**Additional file 1.**

## Data Availability

The datasets used and/or analysed during the current study are available from the corresponding author on reasonable request.
