# Supplementary material for: A quantitative test of the face validity of behavior-change messages based on the Brazilian Dietary Guidelines
Source: Nutr J. 2021 Jan 26;20:10. doi: 10.1186/s12937-021-00668-7 (PMC7839223; doi:10.1186/s12937-021-00668-7)
Supplement: Supplementary file 1 — Additional file 1. [file 12937_2021_668_MOESM1_ESM.docx]

**Supplementary material**

**The behavior-change messages tested in this study**

‘Alimentos *in natura*' são obtidos de plantas ou de animais, e não sofrem qualquer modificação pelo homem. Os chamados 'alimentos minimamente processados' passam por processos como limpeza, fermentação, pasteurização ou congelamento, mas sem interferir na sua qualidade. Esses alimentos não recebem adição de sal, açúcar, óleos, gorduras, nem outros ingredientes.

Torne 'alimentos *in natura*' e 'alimentos minimamente processados' a base de todas as suas preparações culinárias! Cada vez mais brasileiros adotam essa prática, pois existem tantos grãos (cereais e feijões), legumes, verduras e frutas frescas para escolher que basta fazer as combinações.

Quanto mais sabores, cores e texturas em seu prato, mais variada e equilibrada será a sua refeição!

Alimentos 'processados' são alimentos que recebem sal, açúcar, vinagre ou óleo para durarem mais tempo. São exemplos de alimentos processados: pães simples (feitos com farinha, sal, fermento e água), queijos, compotas (frutas em calda), frutas cristalizadas, conservas de legumes, extratos de tomate, pescados entalados (sardinha) e secos (bacalhau), carnes salgadas ou defumadas (carne seca, toucinho).

Os 'alimentos processados' são ricos em sal, açúcar e gordura. Procure usá-los apenas como ingredientes em preparações com 'alimentos *in natura*' e 'alimentos minimamente processados'. Exemplo: milho enlatado adicionado em salada de folhas verdes; queijo ralado adicionado ao macarrão; e carne seca em um escodindinho de mandioca.

Os 'alimentos ultraprocessados' são uma mistura de vários ingredientes que não existem na sua cozinha. É comum lermos nos rótulos desses produtos palavras como 'proteína de soja', 'extrato de carne', 'gordura vegetal hidrogenada', 'xarope de frutose', espessante, emulsificante, corante, aromatizante, realçador de sabor...

Cada vez mais brasileiros evitam 'alimentos ultraprocessados'. Devemos restringir seu consumo, pois os meios de produção, distribuição, comercialização e uso causam danos ao meio ambiente e interferem na vida social e na preservação da cultura alimentar.

O consumo diário de 'alimentos ultraprocessados' aumenta as chances de desenvolver diabetes e hipertensão. Evite-os!

Evite usar temperos ultraprocessados como caldos de carne, frango ou legumes (tablete ou pó) nas suas receitas, pois contêm muito sódio e gordura! Prefira as ervas frescas ou secas e especiarias, como coentro, salsa, cebola, louro, pimenta, orégano, açafrão, além de cebola, alho e pouco sal.

Planeje-se para comprar e cozinhar a sua comida. Lembre-se: planejar é economizar tempo, dinheiro e esforço. Faça uma lista dos alimentos da geladeira e da despensa e organize-os por data de validade (quanto mais próximo ao vencimento, mais rápido devem ser consumidos). Isso ajudará a definir o cardápio da próxima semana e a economizar tempo e dinheiro. Organize-se para cozinhar o suficiente para mais de uma refeição. Congele parte da comida para usar ao longo da semana. Isso facilitará a sua vida!

Organizar a despensa, preparar a lista de compras, fazer as compras, cozinhar, lavar e secar a louça... Todas as tarefas devem ser divididas entre os integrantes da família. Ninguém deve ficar sobrecarregado.

Quando for às compras, leve uma lista com os ingredientes que você precisará para o preparo das refeições e lanches da família. Isso lhe ajudará a evitar comprar alimentos que você não serão usados ou fazer escolhas por influência das propagandas presentes no local. Cuidado com as compras por impulso! Já fez as suas listas? Então, às compras! Visite mercados locais, feiras livres ou de produtores e outros espaços onde possa comprar diretamente deles. Ali, você encontra alimentos frescos e de época a preços baixos e também colabora para proteger os recursos naturais e a biodiversidade.

Priorize alimentos frescos que sejam de época e da região. Além de mais saborosos e de menor custo, ajudam na economia local e contribuem para reduzir o nível de poluição gerada com o transporte.

O que tem na geladeira? Vamos cozinhar! Assar, grelhar, refogar, existem muitas maneiras de preparar comidas deliciosas e saudáveis. Prepare a sua! Se você tem dificuldade para cozinhar, busque ajuda dos amigos e da família. E lembre-se: você também pode aprender a preparar receitas obtidas de livros e na internet.

Cozinhar pode ser muito prazeroso! Pratique essa habilidade com amigos ou membros da família, pois servirá de incentivo para começar. Incluir seus filhos nessa brincadeira pode estimulá-los a gostar de cozinhar!

Torne agradáveis e prazerosos os momentos de preparar e fazer as refeições!

Para economizar tempo, alguns pratos que demandam mais horas de preparo, como o feijão, podem ser feitos em quantidades maiores e congelados para uso durante a semana.

Procure fazer sua própria comida. Evite comer alimentos pré-prontos, que basta abrir e aquecer direto da embalagem. Existem diversas variedades de alimentos frescos para escolher! Experimente receitas diferentes e explore o que sua região tem a oferecer! As verduras e os legumes são os alimentos mais fáceis de incorporar às refeições. Podem ser preparados de diversas formas: crus, assados, refogados, cozidos. Experimente-os em saladas, sopas, ou como complemento ao arroz com feijão! Dê uma segunda chance aos vegetais! Experimente novamente aqueles grãos, legumes, verduras ou frutas que você não gosta, procurando um modo diferente de prepará-los.

Modere na carne vermelha e dê preferência a aves, pescados e ovos. Procure fazer preparações grelhadas, cozidas ou assadas.

Evite substituir preparações culinárias feitas com alimentos (arroz, feijão, verduras e legumes, macarrão, carnes, sopas, molhos e tortas) por produtos prontos ou semi-prontos que não exigem preparações (pratos congelados pré-prontos, macarrão instantâneo, salsichas e outros embutidos, sopa instantânea, molhos industrializados, misturas para bolos).

Abuse dos legumes e verduras! Já as carnes vermelhas não precisam fazer parte de todas as refeições. Dê preferência a carnes brancas ou cortes com pouca gordura e a preparações assadas ou grelhadas.

Procure substituir temperos prontos ou industrializados por alho, cebola, ervas e especiarias, como louro, salsa, pimenta, orégano, alecrim, cominho e açafrão-da-terra, para melhorar o sabor e os benefícios à saúde. E modere na quantidade de sal e óleo que você coloca nas preparações.

Frutas são lanches perfeitos! Frescas ou secas, elas podem fazer parte de qualquer refeição (em saladas ou como sobremesa), mas são especialmente bem-vindas no café da manhã e nas pequenas refeições. Leite, iogurte natural (não aromatizado e não adoçado), castanhas, farinhas, farelos ou flocos de cereais, como a aveia, podem acompanhar as frutas frescas ou secas das pequenas refeições.

Procure acostumar seu paladar ao sabor natural dos alimentos. Antes de adicionar açúcar ou sal, experimente-os. Evite adoçar sucos, leite, chás ou café e salgar pratos já prontos.

Fazer as refeições sempre nos mesmos horários regula melhor o apetite e você tem menos vontade de "beliscar" entre elas. Planeje com antecedência o que será consumido ao longo do dia, especialmente quando estiver fora de casa.

Fique atento aos sinais de sede! Hidrate-se com água pura ou aromatizada com fatias de frutas frescas ou ervas, como limão, laranja, hortelã. Evite refrigerantes, bebidas energéticas e sucos industrializados, pois possuem alto teor de açúcar e aditivos não benéficos à saúde.

Compartilhe o tempo de refeição com a família, os amigos ou os colegas de trabalho. Sente-se à mesa, sem distrair-se com TV ou celular. Transforme suas refeições em momentos prazerosos!

Transforme suas refeições em momentos prazerosos! Busque comer em locais agradáveis e tranquilos, sente-se à mesa e em companhia, sem distrair-se com TV ou celular.

Ao comer fora de casa, opte por um restaurante limpo e confortável. Dê preferência a locais que sirvam comida por quilo, ou que sirvam pratos à base de arroz, feijão, verduras e legumes: o famoso pê-efe brasileiro! Evite comer em locais que incentivem o consumo ilimitado de alimentos, como rodízios e buffets livres.
